# Updated grading system for systemic allergic reactions: Joint Statement of the World Allergy Organization Anaphylaxis Committee and Allergen Immunotherapy Committee

**DOI:** 10.1016/j.waojou.2024.100876

**Published:** 2024-02-10

**Authors:** Paul J. Turner, Ignacio J. Ansotegui, Dianne E. Campbell, Victoria Cardona, Stuart Carr, Adnan Custovic, Stephen Durham, Motohiro Ebisawa, Mario Geller, Alexei Gonzalez-Estrada, Paul A. Greenberger, Elham Hossny, Carla Irani, Agnes S.Y. Leung, Michael E. Levin, Antonella Muraro, John J. Oppenheimer, José Antonio Ortega Martell, Guillaume Pouessel, Manuel J. Rial, Gianenrico Senna, Luciana K. Tanno, Dana V. Wallace, Margitta Worm, Mário Morais-Almeida, the WAO Anaphylaxis Committee and WAO Allergen Immunotherapy Committee

**Affiliations:** a National Heart Lung Institute, Imperial College London, London, UK; b Dept. Allergy and Immunology, Hospital Quironsalud Bizkaia, Bilbao, Spain; c Discipline of Paediatrics and Child Health, School of Medicine, University of Sydney, Sydney, Australia; d DBV Technologies, Montrouge, France; e Allergy Section, Department of Internal Medicine, Hospital Vall d’Hebron, Barcelona, Spain; f Snö Asthma & Allergy, Abu Dhabi, United Arab Emirates; g Department of Allergy, Clinical Research Center for Allergy and Rheumatology, National Hospital Organization Sagamihara National Hospital, Kanagawa, Japan; h Division of Medicine, Academy of Medicine of Rio de Janeiro, Rio de Janeiro, Brazil; i Division of Allergy, Asthma, and Clinical Immunology, Department of Medicine, Mayo Clinic, Scottsdale, AZ, USA; j Division of Allergy-Immunology, Department of Medicine, Northwestern University Feinberg School of Medicine, Chicago, IL, USA; k Pediatric Allergy and Immunology Unit, Children's Hospital, Ain Shams University, Cairo, Egypt; l Hotel Dieu de France Hospital, St Joseph University, Beirut, Lebanon; m Department of Paediatrics, The Chinese University of Hong Kong, Prince of Wales Hospital, Shatin, Hong Kong; n Division of Paediatric Allergy, Department of Paediatrics, University of Cape Town, Cape Town, South Africa; o Department of Woman and Child Health, Food Allergy Referral Centre, Padua University Hospital, Padua, Italy; p Rutgers New Jersey Medical School, Atlantic Health System Morristown, NJ, USA; q Universidad Autónoma del Estado de Hidalgo, Hidalgo, Mexico; r Department of Paediatrics, Children's Hospital, Roubaix, France; s Paediatric Pulmonology and Allergy Department, Hôpital Jeanne de Flandre, CHU Lille, Lille, France; t Allergy department, Complexo Hospitalario Universitario A Coruña, A Coruña, Spain; u Asthma Center and Allergy Unit, Verona University and General Hospital, Verona, Italy; v Hospital Sírio Libanês, São Paulo, Brazil; w University Hospital of Montpellier, Montpellier, and Sorbonne Universités, Paris, France; x Nova Southeastern University College of Allopathic Medicine, Fort Lauderdale, FL, USA; y Department of Dermatology and Allergology, Charite-Universitätsmedizin, Berlin, Germany; z Allergy Center, CUF Descobertas Hospital, Lisbon, Portugal

**Keywords:** Adverse events, Allergen immunotherapy, Anaphylaxis, Clinical trials, Safety reporting

## Abstract

There is a lack of consensus over the description and severity assignment of allergic adverse reactions to immunotherapy, although there seems to be a consensus at least in terms of using the World Allergy Organization (WAO) grading systems to describe local adverse events for Sublingual Immunotherapy (SLIT) and Systemic Allergic Reactions (SARs) to Subcutaneous Immunotherapy (SCIT) amongst the major national/regional allergy societies. In this manuscript, we propose a modification of the previous WAO Grading system for SARs, which aligns with the newly-proposed Consortium for Food Allergy Research (CoFAR) Grading Scale for Systemic Allergic Reactions in Food Allergy (version 3.0). We hope this can facilitate a unified grading system appropriate to SARs due to allergen immunotherapy, independent of allergen and route of administration, and across clinical and research practice.

## Introduction

Assessment of allergic adverse events and assignment of reaction severity is important in clinical practice, and it is also a key element of clinical trials monitoring and post-approval safety reporting. However, numerous severity grading systems for allergic reactions have been described in the literature.^1^^,^^2^ Many of these systems were originally developed to grade reactions of a specific allergen type (eg, venom) and are not necessarily appropriate when applied to other allergen types. For example, the grading systems proposed by Ring and Messmer^3^ and Mueller^4^ were originally intended to grade allergic reactions to drug- and venom-induced reactions respectively. Both have been applied to grade severity of food-induced reactions, but this is inappropriate as they assign a greater severity to vomiting as a symptom, which is far more significant in reactions caused by non-food triggers.^5^ Unfortunately, there is no consensus with respect to the most appropriate system to grade allergic adverse events.^6^

A number of comparisons of the different grading systems have been published, which highlight the differences and relative deficiencies of each system, particularly when applied to a different allergen than originally intended.^2^^,^^7^^,^^8^ Furthermore, existing grading systems may not optimally assign severity of allergic adverse reactions to allergen immunotherapy, for example due to lack of granularity or over-reliance on subjective judgments by investigators (Table 1). The situation is further complicated by the inconsistent application of different clinical criteria to define anaphylaxis in both research and clinical practice.^5^

**Table 1:** Stakeholder perceptions of severity. Adapted from Stafford et al.^7^

Stakeholder	Perception of severity and possible implications
Patients with allergies and their caregivers	May underestimate or overestimate severity: parents of children with food allergies may perceive significant skin signs (eg, facial angioedema) as severe, whereas experienced clinicians recognize that this is a common presentation of reactions in young children.In contrast, parents may attribute wheeze to a viral illness (particularly in a child prone to viral wheeze) and fail to recognize that this indicates anaphylaxis if occurring after exposure to a known allergen.
Non-allergy specialist healthcare professionals including emergency department staff	Need to consider long lists of differential diagnoses.May have limited experience with anaphylaxis, leading to inaccurate or delayed diagnosis, or inappropriate treatment (possibly linked to reluctance to administer epinephrine). Reactions may have resolved by arrival to hospital, so severity assignment in hospital may not reflect true reaction severity.
Allergy specialists	Trained to evaluate the spectrum of allergic disease, often by retrospective assessment of severity on the basis of patient or parent-report. Often not involved in the provision of acute care in Emergency departments or clinical trials.
Regulatory bodies	Necessity for objective assessment, since severity assessment may be performed by non-allergy specialists. Severity may also, in practice, be informed by whether reaction has resulted in an unscheduled health encounter. Thus, mild reactions presenting to hospital might be classified as more severe than is anaphylaxis managed in the community.

To address some of these limitations with respect to assignment of severity in the context of Subcutaneous Immunotherapy (SCIT), in 2009, a World Allergy Organization (WAO) International Task Force (which included representatives from regional and national allergy societies, various international health care organizations, and the National Institute of Allergy and Infectious Diseases [NIAID]) proposed a new schematic (WAO Subcutaneous Immunotherapy Systemic Reaction Grading System) which was published in 2010^9^ and endorsed by the American Academy of Allergy, Asthma & Immunology (AAAAI), the Latin American Society of Allergy and Immunology (SLAAI), the Asia Pacific Association of Allergy, Asthma and Clinical Immunology (APAAACI), and the American College of Allergy Asthma and Immunology (ACAAI).

The 2010 WAO Grading System has not only been used in the context of reactions to SCIT but also for sublingual immunotherapy (SLIT).^10^ In response to this trend, a modification was proposed in 2016 to allow its application to grade Systemic Allergic Reactions (SARs) irrespective of trigger.^10^ This grading system was subsequently incorporated into the 2020 WAO Anaphylaxis Guidance document,^5^ which was endorsed by over 50 national allergy societies. However, the WAO grading system for SARs has not, in general, been applied to studies of allergen immunotherapy (AIT) for food allergy. Rather, these studies have often utilized a grading system developed by the Consortium for Food Allergy Research (CoFAR), published in 2012.^11^ However, as reported by the CoFAR investigators, the United States Food and Drug Administration (FDA) determined that the previous scheme was too subjective for widescale use.^12^ In response, in 2022, CoFAR published a new grading system that incorporated objective signs/symptoms (the Grading Scale for Systemic Allergic Reactions in Food Allergy v3.0),^12^ with a similar format to the WAO SAR Grading System.

## Updated WAO grading system for systemic allergic reactions

**Fig. 1:**
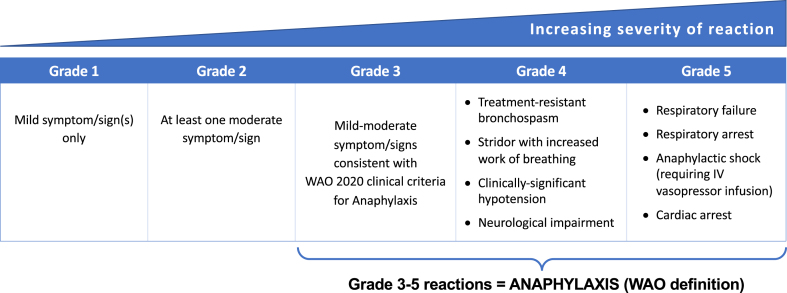
Concept underlying the WAO Grading system

Given the similar formats of the modified WAO and CoFAR systems, the WAO Anaphylaxis Committee undertook a mapping exercise to align the CoFAR scale with the WAO SAR Grading system, with the objective to achieve a system which would be suitable and appropriate for use across all clinical trials and in post-surveillance studies to grade SARs irrespective of cause (SCIT, SLIT, oral immunotherapy [OIT], intra-lymphatic, etc.) and implicated allergen. In addition, the grading system could also be applied to clinical and cohort studies that do not involve AIT. The concept is shown in Fig. 1, and the new WAO Grading System for Systemic Allergic Reactions is presented in Table 2.

**Table 2:** Updated WAO Grading system for systemic allergic reactions.

Grade 1	Grade 2	Grade 3	Grade 4	Grade 5
Mild symptom/sign(s) only: NB: Reactions can be further categorized as: • **1t**: transient (<20 mins) • **1s**: single organ system only, ≥20 mins • **1m**: 2+ organ systems, ≥20 mins	Any 1 (or more) of the following moderate symptom/sign(s):	Any 1 (or more) of the following symptom/signs:	Any 1 (or more) of the following symptom/signs:	Any 1 (or more) of the following symptom/signs:
***Cutaneous****(any one of):* • Limited (few) or localized hives/urticaria^a^ • Skin flushing (few areas of faint erythema) or mild pruritus^a^ • Swelling (e.g. lip edema)^a^ ^a^*excluding localized symptoms at application site*	***Cutaneous****(any one of):* • Systemic urticaria (e.g. numerous or widespread hives) • Generalized (≥50% BSA) erythema • Widespread pruritus with protracted scratching • Significant angioedema (excluding lip swelling and laryngeal edema)	***Lower respiratory*** • Bronchospasm (e.g. wheezing, shortness of breath) which responds to first line treatment • Cough due to laryngeal or lower respiratory involvement	***Respiratory*** • Severe bronchospasm (not improving with 2 doses of IM epinephrine ± other appropriate treatment) • Stridor (with increased work of breathing)	***Respirator*** • Respiratory failure requiring positive pressure ventilation • Respiratory arrest
Or	And/or	And/or	And/or	And/or
***Upper respiratory*** • Nasal symptoms (e.g., sneezing, rhinorrhea, itch, congestion) • Throat-clearing (itchy throat)^a^ or throat tightness/discomfort • Cough due to throat irritation or nasal symptoms		***Upper respiratory/laryngeal*** • Throat tightness with vocal hoarseness • Stridor without increased work of breathing • Persisting (≥20 mins) odynophagia (pain on swallowing)	***Cardiovascular*** • Hypotension with associated symptoms of end-organ dysfunction (e.g. hypotonia, dizzy^§^, collapse^§^, syncope) OR • decrease in systolic blood pressure (sysBP) ≥30% from that person's baseline OR • SysBP <90 mmHg in adults (in children ≤10 years, sysBP <70 mmHg + [2 × age in years]) ^*§*^*excluding vasovagal events (these present with dizziness/fainting which rapidly resolve on lying flat)*	***Cardiovascular*** • Anaphylactic shock i.e. requirement for IV vasopressor infusion to maintain sysBP ≥90 mmHg or MAP ≥65 mmHg in adults and children >10 years (or age-appropriate sysBP in younger children) • Cardiac arrest
Or		And/or		
***Gastrointestinal*** • Nausea • Mild abdominal pain (for example, without a change in activity level)	***Gastrointestinal*** • Persisting (≥20 mins) and non-distractable abdominal pain and/or • Vomiting (not due to gag or taste aversion) and/or diarrhea	***Gastrointestinal AND Cutaneous*** Severe GI symptoms together with cutaneous features which meet WAO 2020 criteria for anaphylaxis (e.g. severe crampy abdominal pain, repetitive vomiting, *especially after exposure to a non-ingested allergen)*		
Or		And/or	And/or	
***Other*** • Conjunctival reddening (not due to eye rubbing), pruritus, or tearing • Metallic taste		**Uterine** cramps ± uterine bleeding	***Neurological*** • *Glasgow Come Scale* < *13*	

Grade 2 reactions are not usually considered anaphylaxis according to WAO 2020 clinical criteria, but may respond to treatment with epinephrine.

BSA, body surface area; sysBP, systolic blood pressure; MAP, mean arterial pressure.

a Application-site reactions are considered local reactions, see text for more information on classification of local reactions

Of note, both the previous WAO and CoFAR systems assign Grade 2 severity as either mild symptoms in 2 or more organ systems, or *any* moderate symptoms. However, this can lead to the scenario where mild symptoms from 2 different organ systems (for example, mild rhinitis and mild erythema) would be categorized as the same severity as widespread generalized urticaria or persistent and non-distractable abdominal pain with vomiting. To avoid this scenario, the workgroup voted to change Grade 2 reactions to moderate reactions only (85% agreement amongst the group). A further change was also proposed whereby mild symptoms from more than 1 organ system can still be distinguished from single-organ involvement (see Considerations Relating to Data Collection, below).

In order to align the 2 systems, the following modifications were made:

1. In line with the 2010 WAO grading system, Grade 5 reactions incorporate the most severe SARs (cardiovascular and respiratory arrest), which by definition also includes “death” (which equates to Grade 5 reactions in the CoFAR scale). This allows for greater discrimination of more severe (but not life-limiting) allergic reactions across 3 grades rather than just 2.
2. A single episode of vomiting or diarrhea was considered a Grade 1 sign in CoFAR, while all gastrointestinal symptoms were grade 2/3 in the 2010 WAO system. The Committee reviewed the differences between the 2 systems (Table 3) and agreed to assign gastrointestinal symptoms/signs as Grade 2, with the exception of mild subjective gastrointestinal symptoms (assigned as Grade 1) and severe gastrointestinal symptoms which meet the WAO criteria for anaphylaxis^5^ as Grade 3. Where a single episode of vomiting or diarrhea occurs without systemic features, in the context of exposure via the oral route (for example, due to OIT or SLIT), this is considered a local rather than systemic reaction. In contrast, any objective gastrointestinal sign resulting from systemic exposure (ie, the non-oral route) should always be considered at least Grade 2 severity.

**Table 3:** Severity assignment of gastrointestinal symptoms/signs

	Grade 1	Grade 2	Grade 3
WAO SAR grading system (2016)	–	• Abdominal cramps and/or • Vomiting/diarrhea that do not meet WAO criteria for anaphylaxis	• Abdominal cramps and/or • Vomiting/diarrhea
CoFAR v3 (2022)	Nausea, abdominal pain (no change in activity level), single episode of vomiting and/or single episode of diarrhea	Nausea, abdominal pain (with change in activity level), two episodes of vomiting and/or diarrhea	Severe abdominal pain, more than two episodes of vomiting and/or diarrhea
Updated WAO SAR grading system (2023)	• Nausea • Mild abdominal pain without a change in activity level	• Persisting (≥20 mins) and non-distractable abdominal pain, and/or • Vomiting (not due to gag or taste aversion) and/or diarrhea	• Severe and persisting (≥20 mins) abdominal pain, and/or • Repetitive vomiting Especially following exposure to non-food allergens
3. In line with CoFAR, stridor without increased work of breathing is classified as Grade 3, while stridor with increased work of breathing remains as Grade 4.
4. While use of *any* epinephrine (adrenaline) is not a good indicator of severity due to variations in prescribing practice, definitions of anaphylaxis and suboptimal use in proven anaphylaxis, there is increasing recognition that a suboptimal response (ie, ongoing Airway/Breathing/Circulation symptoms of anaphylaxis) to 2 appropriate doses of intramuscular (IM) epinephrine can be a useful indicator of severity.^13^ Such an approach circumvents issues over what symptoms constitute a severe reaction (and according to which definition or grading system), since less severe reactions would not be resistant to epinephrine treatment. On the basis that bronchospasm occurring due to an immunotherapy dose is anaphylaxis and must always be treated with IM epinephrine (as per international guidelines), the Committee agreed that Grade 4 respiratory reactions should be defined by bronchospasm which fails to improve with two doses of IM epinephrine, as this also provides a degree of objectivity in assessing severity.^13^ Hypoxemia is not included as a defining feature, as it is assumed that oxygen would be administered in the context of a severe reaction and thus confound the assessment of hypoxemia in room air.
5. Historically, significant cardiovascular involvement (eg, hypotension) has been categorized as the maximum non-fatal grade (grade 5 in 2010 WAO system, grade 4 in CoFAR). Arguably, hypotension may respond rapidly to initial epinephrine and should not necessarily be considered as more severe than refractory bronchospasm. On this basis, significant cardiovascular features (either *any* hypotension with end-organ dysfunction, or significant hypotension alone) have been categorized as Grade 4, while anaphylactic shock (defined according to Dribin et al^14^) and cardiac arrest remain as Grade 5.
6. With increasing recognition that neurological symptoms can occur without obvious systemic hypotension (and likely to be due to the effect of inflammatory mediators (including histamine) within the central nervous system (CNS) and/or local CNS perfusion), a fall in Glasgow Coma Scale <13 (a cut-off proposed by Dribin et al^14^) has been classified at Grade 4. Severe neurological involvement is likely to be secondary to systemic hypotension and is therefore not separately categorized.

## Assessment of local (rather than systemic) allergic reactions

Consistent with the 2010 WAO grading system and the CoFAR scale, application-site reactions should be considered local, rather than systemic reactions. Examples of local reactions would therefore include:

• oral mucosal symptoms (eg, oral pruritus, itchy throat) after sublingual (SLIT) or oral immunotherapy (OIT)
• gastrointestinal symptoms after SLIT or OIT, in the absence of systemic manifestations
• warmth and/or pruritus at an injection site for subcutaneous immunotherapy or vaccine administration
• skin reactions at the site of allergen application with epicutaneous immunotherapy (EPIT)

A grading system has been published for local adverse reactions to SLIT.^15^ In addition, a modified grading system based on a European Task Force on Atopic Dermatitis (ETFAD) scheme for atopy patch test reading has been used in FDA-regulated clinical trials for EPIT.^16^ No equivalent system has been published for local adverse reactions to SCIT or OIT. For SCIT, it is important to distinguish between local reactions (LR) and large local reactions (LLR). LRs are defined as swelling and redness that occur in the immediate vicinity of the injection site, and can cause pain, localized edema, and (sometimes itchy) erythema.^17^ Cut-offs for LLRs are variably defined in the literature, ranging from 20 to 25 mm to over 10 cm.^17^ For venom immunotherapy, an EAACI taskforce has defined an LLR as “a swelling exceeding a diameter of 10 cm that lasts for more than 24 hours.”^18^ The current consensus seems to be to define an LLR as “redness/swelling >10 cm in diameter,”^19^ although this seems to be based on previous studies which define LLR as an area of redness/swelling greater than the size of the patient's palm (which in an adult is around 8–10 cm).^20^

There is no current consensus over the assignment of non-systemic (ie, local) adverse events for OIT. One approach has been to use a similar scheme to that for SARs, but flag when such reactions are local and/or transient (self-resolving within 20 min).^21^ Alternatively, the WAO grading system for SLIT could easily be applied to OIT. These approaches are summarized in Table 4.

**Table 4:**
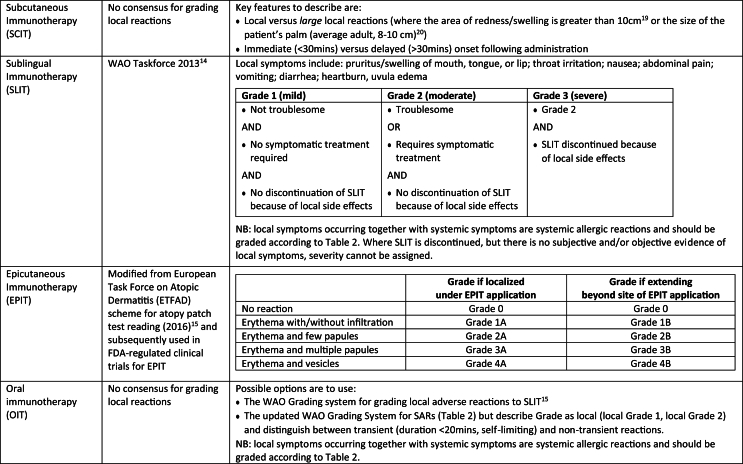
Summary of mainstream approaches to describe/grade local adverse reactions to immunotherapy

## Considerations relating to data collection

The Committee For Medicinal Products For Human Use (CHMP) of the European Medicines Agency has recommended that “expected allergic adverse events should be distinguished into immediate or delayed effects, according to the time of appearance (immediate when the onset of the reaction is during the first 30 min after the administration and delayed when the onset is after the first 30 min of the administration) and into local and systemic effects according to the site of the appearance of the reaction (local when the reaction takes place in the administration site and systemic when the reaction takes place far from the administration site) and reported separately”.^22^

The original 2010 WAO Systemic Allergic Reaction (SAR) Grading System^9^ proposed that data collection relating to both systemic and local adverse reactions should include the following additional information:

• the first symptom(s)/sign(s)
• time of onset after allergen administration
• timing and amount of epinephrine, if administered.

With the change to Grade 2 reactions being limited to only moderate symptoms, the working group proposed that Grade 1 reactions could be further sub-categorized into:

• Grade **1t** (transient), where symptoms completely resolve within 20 min
• Grade **1s** (single organ system), where mild symptoms persist ≥20 min and involve just a single organ system
• Grade **1m** (multiple organ system), where mild symptoms persist ≥20 min and involve more than a single organ system

## Management of SARs

Importantly, this new aligned Grading System is not meant as a tool to guide treatment of reactions. While Grade 3–5 reactions are aligned with the current WAO clinical criteria for anaphylaxis (and thus should be treated with IM epinephrine),^4^ this does not imply that Grade 2 (or even Grade 1) systemic reactions are *not* be treated with epinephrine – something particularly important in the context of venom immunotherapy. For example, acute and progressing generalized urticaria following SCIT injection should be treated with IM epinephrine (and certainly not with antihistamine alone). Within the author group, there is anecdotal experience that palmar itch often precedes severe anaphylaxis in the context of SCIT, as well as with food-dependent, exercise-induced anaphylaxis; patients with these symptoms should justifiably be treated with IM epinephrine early.

## Summary

There is still a relative lack of consensus over the description and severity assignment of allergic adverse reactions to immunotherapy, although there seems to be a consensus at least in terms of using the WAO grading systems to describe local adverse events for SLIT and SARs to SCIT amongst the major national/regional allergy societies (including EAACI,^19^ AAAAI,^9^ ACAAI^9^). We propose a modification of the previous WAO Grading system for SARs, which aligns with the newly-proposed CoFAR Grading Scale for Systemic Allergic Reactions in Food Allergy (version 3.0). While designed primarily to describe SARs due to allergen immunotherapy irrespective of route of administration, the scheme can also be applied to reactions occurring due to accidental exposure and in clinical practice. We hope this can facilitate a unified grading system appropriate to SARs, independent of allergen, across clinical and research practice.

## Abbreviations

AAAAI, Academy of Allergy, Asthma & Immunology; ACAAI, American College of Allergy Asthma and Immunology; APAAACI, Asia Pacific Association of Allergy, Asthma and Clinical Immunology; BSA, body surface area; CHMP, Committee For Medicinal Products For Human Use; CoFAR, Consortium for Food Allergy Research; EPIT, Epicutaneous immunotherapy; FDA, United States Food and Drug Administration; LLR, large local reactions; LR, local reactions; MAP, mean arterial pressure; OIT, Oral immunotherapy; SAR, Systemic Allergic Reaction; SCIT, Subcutaneous Immunotherapy; SLAAI, Latin American Society of Allergy and Immunology; SLIT, Sublingual Immunotherapy; sysBP, systolic blood pressure; WAO, World Allergy Organization.

## Funding

Not applicable.

## Availability of data and materials

Not applicable.

## Author contributions / Consent for publication:

PJT led the drafting of the initial manuscript, which was then circulated to all authors who subsequently provided input into the manuscript, reviewed the final draft and provided consent for publication.

## Ethics approval

Not applicable.

## Declaration of competing interest

All authors have completed the ICMJE uniform disclosure form at and declare no funding for the submitted work. P.J. Turner reports grants from the UK Medical Research Council, UK Food Standards Agency, The Jon Moulton Charity Trust, NIHR/Imperial Biomedical Research Centre and End Allergies Together, outside the submitted work; personal fees from UK Food Standards Agency, DBV Technologies, Aimmune Therapeutics, ALK, Allergenis and ILSI Europe outside the submitted work. D.E. Campbell is employed by DBV Technologies. S. Carr reports personal fees from Sanofi and Biologix, outside the submitted work. A. Custovic reports personal fees from Novartis, Sanofi, Stallergenes Greer, AstraZeneca, Worg Pharmaceuticals and GSK, outside the submitted work. S. Durham reports nonfinancial support from ALK and personal fees from ALK, Stallergenes Greer, Revelo, and ANGANY, Inc, outside the submitted work. M. Ebisawa reports personal fee from Viatris outside the submitted work. AM reports personal fees from Viatris, Aimmune, DVB Technologies, Sanofi Regeneron, Nestlè Health Science, outside the submitted work. M. Levin declares consulting fees from Impulse Biomedical, outside the submitted work. J.J. Oppenheimer reports personal fees from Aquestive and ARS, outside the submitted work. G. Pouessel declares personal fees from Bausch & Lomb, Meda/Mylan/Viatris, Stallergenes Greer, Novartis, ALK-Abello, DVB Technologies, AImmune Therapeutics, Theravia outside the submitted work. D.V. Wallace reports personal fees from Bryn and ARS outside the submitted work. The other authors do not report any conflicts of interest.
